# Neighbour–stranger discrimination in an African wood dove inhabiting equatorial rainforest

**DOI:** 10.1038/s41598-024-53867-7

**Published:** 2024-02-21

**Authors:** Małgorzata Niśkiewicz, Paweł Szymański, Lia Zampa, Michał Budka, Tomasz S. Osiejuk

**Affiliations:** Department of Behavioural Ecology, Institute of Environmental Biology, Faculty of Biology, Adam Mickiewicz University, Uniwersytetu Poznańskiego 6, 61-614 Poznań, Poland

**Keywords:** Neighbour–stranger discrimination, Dear enemy phenomenon, Broadcast vocalisation, Song, Columbidae vocalisation, Playback experiment, Animal behaviour, Behavioural ecology, Tropical ecology, Ecology, Zoology

## Abstract

We investigated within- and between-individual song variation and song-based neighbour-stranger discrimination in a non-learning bird species, the blue-headed wood-dove (*Turtur brehmeri*), which inhabits lowland rainforests of West and Central Africa. We found that songs of this species are individually specific and have a high potential for use in individual recognition based on the time–frequency pattern of note distribution within song phrases. To test whether these differences affect behaviour, we conducted playback experiments with 19 territorial males. Each male was tested twice, once with the songs of a familiar neighbour and once with the songs of an unfamiliar stranger. We observed that males responded more aggressively to playback of a stranger’s songs: they quickly approached close to the speaker and spent more time near it. However, no significant differences between treatments were observed in the vocal responses. In addition, we explored whether responses differed based on the song frequency of the focal male and/or that of the simulated intruder (i.e., playback), as this song parameter is inversely related to body size and could potentially affect males’ decisions to respond to other birds. Song frequency parameters (of either the focal male or the simulated intruder) had no effect on the approaching response during playback. However, we found that the pattern of response after playback was significantly affected by the song frequency of the focal male: males with lower-frequency songs stayed closer to the simulated intruder for a longer period of time without singing, while males with higher-frequency songs returned more quickly to their initial song posts and resumed singing. Together, these results depict a consistently strong response to strangers during and after playback that is dependent on a male’s self-assessment rather than assessment of a rival’s strength based on his song frequency. This work provides the first experimental evidence that doves (Columbidae) can use songs for neighbour-stranger discrimination and respond according to a “dear enemy” scheme that keeps the cost of territory defence at a reasonable level.

## Introduction

The ability to recognise one individual from another based on distinctive features has evolved many times across different animal taxa^1^ using different signal modalities^2^. In birds, acoustic signals like songs or calls often have individually distinct characteristics that are used for individual recognition in a wide range of contexts, such as parent–offspring recognition, social behaviours (foraging, anti-predator, roosting, etc.), or neighbour-stranger discrimination (hereafter NSD)^3–5^. NSD in birds is strongly supported by research^5,6^ and has a well-recognised ecological and evolutionary background^2^. Many bird species defend access to limited resources such as territories, mates, or food, which yields evolutionary benefits in terms of increased fitness. However, defending resources is inherently linked with costs related to signalling, patrolling, and chasing intruders, which may increase energy expenditure, the risk of predation, or even the risk of injury or death caused by a rival. Therefore, territoriality should only be observed if, on average, the benefits from limiting access to resources exceed the costs of their defence^7^. The cost of territorial defence can be reduced by avoiding unnecessary conflict, and one way of doing this is by discriminating between familiar neighbours and unfamiliar strangers^6^. Neighbours do not necessarily constitute a serious threat to a territory holder, whereas the appearance of a stranger carries the risk of territorial insertion, takeover, or interception of a female. Therefore, after a territory's borders are established, the response of the territory holder to an intrusion by a familiar neighbour should be less aggressive than the reaction to an intrusion by a stranger. This reduction in aggression toward neighbours has been termed the "dear enemy phenomenon"^8^ and has been observed in many territorial birds, as well as mammals, reptiles, amphibians, and insects^6,9,10^. However, a “dear enemy” relationship can be flexible and may evolve with the social and ecological circumstances at hand^11,12^. In certain cases, a neighbour can actually be more threatening than a stranger^13,14^. For example, the song sparrow males adjust behaviour towards neighbours based on their own mate’s fertility status and respond stronger to neighbours during periods of female fertility^15^.

NSD in a territorial-defence context has been studied primarily in songbirds (Oscines), i.e., birds that acquire a song through social learning during ontogeny (Stoddard 1996). Although it has rarely been directly expressed, the general opinion seems to be that birds who learn their songs should be better at recognition tasks^16^. Indeed, several studies have reported that individual recognition in songbirds is not limited by characteristics of their repertoires (e.g., large or shared between males)^17,18^. Less is known about NSD in non-learning bird species, but increasingly, experimental evidence is arriving from taxa as varied as grouses^19^, tyrant flycatchers^20^, shearwaters^21^, loons^22^, gulls^23^, woodhoopoes^24^, owls^25^, and rails^26^. To our knowledge, though, one group of birds that has never been tested with respect to NSD is the family Columbidae. Members of this family are often characterised by stereotyped broadcast signals (referred to as both songs and calls), which may create the impression that there is little space for identity coding. On the other hand, in-depth studies of the mechanisms of vocal production^27,28^ and the functions of various characteristics of their voices^29–33^ have shown great communicative abilities. Indeed, many pigeons’ and doves’ songs seem to be aimed at individuals who are far out of sight, and there is evidence that these songs may contain a great deal of individually distinct information^34,35^.

**Figure 1:**
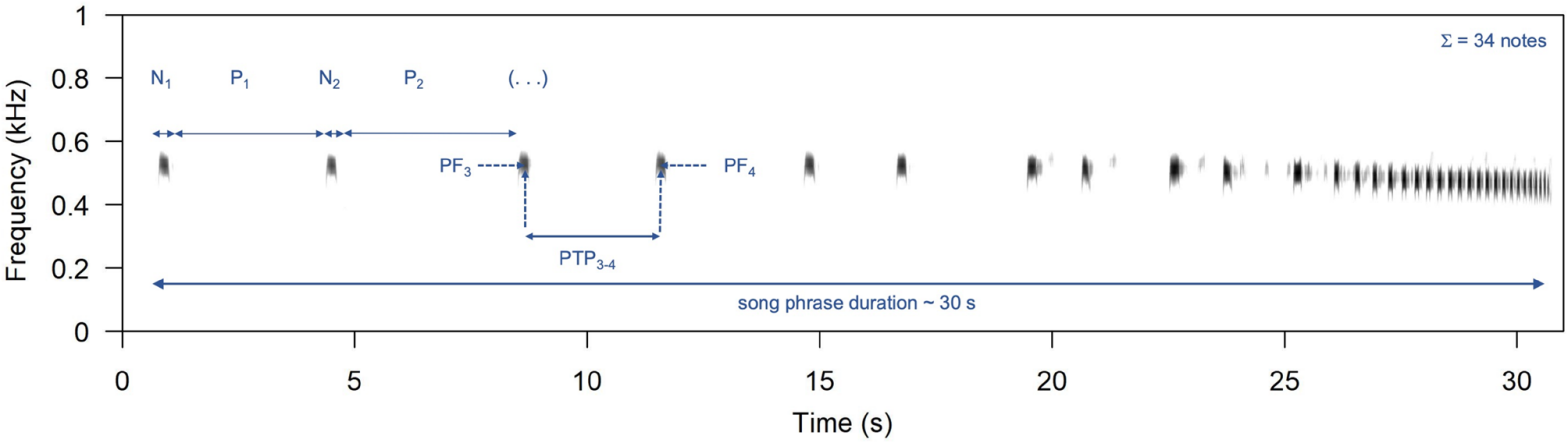
Spectrogram illustrating the song of the blue-headed wood-dove. Letters indicate: N_1_, N_2_—durations of following notes; P_1_, P_2_—pauses between following notes, based on manually selected beginning and end of each note; PF_3_, PF_4_—peak frequency of notes measured with the One-dimensional transformation function of Avisoft SASLab Pro; PTP_3–4_—pauses between syllables measured as time duration between peak frequency of following notes. The figure shows example measurements that were taken for all notes and pauses within each song.

Here, we investigated song-based NSD in the blue-headed wood-dove, *Turtur brehmerii* (Columbidae), in its natural environment. We used the term “song” to describe the signal produced by males, serving both to attract mates and to compete with conspecific rivals. Hence, it possesses crucial song characteristics^36^. Blue-headed wood-doves are non-learning, sedentary birds that inhabit lowland rainforest (up to 750 m asl) in western and central sub-Saharan Africa, where visual contact is difficult. The biology of blue-headed wood-doves has not been well characterised, but males are known to defend their territories and occupy the same area for long periods, presumably their whole lives^37^. Males sing spontaneously from treetops, and the song posts of different individuals are typically separated by ≥ 100–150 m. The breeding season is long and likely depends more on food availability than weather conditions^37^. Territorial blue-headed wood-dove males produce a moderately loud song (79–85 dBA SPL at 1 m) consisting of short whistle syllables of increasing rate (Fig. 1). Despite its loudness, the low frequency of the song (with a peak average frequency of 460 Hz; more details in Table S1) and the unmodulated whistles ensure efficient transmission through the forest habitat. Songs can be heard by human observers even from 400 to 500 m (personal observations). The function of this song appears to be equivalent to the function of song in songbirds, i.e., mate attraction and territory defence^38^. Unlike the songs of many songbirds, though, the blue-headed wood-dove song is seemingly very simple in structure*.*

In this study, we performed the first detailed analysis of basic song parameters in the blue-headed wood-dove and identified which song characteristics might have the potential for identity coding. Then, we used playback to experimentally assess the ability of males of this species to discriminate between the songs of familiar neighbours and unfamiliar strangers, by analysing the behaviour of territory owners during simulated intrusions by neighbours and strangers.

We predicted that if these birds are able to discriminate between neighbours and strangers, we should observe differential responses to the playback of songs from these two groups. Given the sedentary life style and long-term territorialism of blue-headed wood-doves, we expected to observe a stronger response to strangers’ songs. Our parallel study on this species demonstrated that the song of a given male has an extremely repeatable frequency, which reflects body size (negative correlation between song pitch and body size) and may potentially influence the response to a rival (in prep.). Therefore, we also performed additional analyses to determine if the song frequency of the focal males and/or that of the playback songs affected the responses to the simulated neighbours and strangers.

## Methods

### Study area

Our study was conducted in Kakum NP in Ghana (5.20–5.40 N, 1.30–1.51 W, altitude 135–250 m asl). The study area was regularly searched and all individuals recorded and mapped. Then, territorial males were observed, recorded, mist-netted, and individually marked (colour rings and LifeTags™, Cellular Tracking Technologies) in February–March 2021, and November 2021. Captured birds of the study species, as well as the co-occurring sibling species (tambourine dove, *Turtur tympanistria*), were weighted and measured in a standard manner. The experimental portion of the study was conducted between 13 November and 4 December 2022. In Kakum NP, the blue-headed wood-dove is a common forest species and prefers patches with high trees close to streams. During the experimental period, birds were vocally active and sang intensively from dawn until 11–12 h, depending on the weather conditions. Males tended to cluster; within a cluster, 2–5 males could typically be heard singing from song posts separated by 100–300 m, from which it was not possible to hear birds from other groups. It seems that in preferred habitats the space was fully filled with territories, and neighbours singing at distances ≥ 100 m from each other did not approach each other.

### Recording procedures

We recorded males during preliminary fieldwork and during the experimental part of the study in order to obtain good samples for playback preparation. To determine if the songs of different males have the potential for identity coding, we selected high-quality recordings with at least ten songs of each male. To ensure that we analysed only one individual and his songs at a time, we chose well-spaced males and recorded from a short distance, from a single song post (no movements during recording), and in a single attempt. This limited the sample size to 10 males and 200 songs but allowed for unequivocal identification and characterisation of within-individual song variation. We also followed three individuals with mentioned earlier LifeTags; they were recorded while a second observer simultaneously identified them with a radio receiver, in order to confirm that the songs of individuals were constant in time (subsequent days). Here we present a brief overview of the song characteristics of these birds as they relate to NSD; a more detailed comparison of within- and between-individual song variation in all five *Turtur* species will be the subject of a separate study (in prep.). Recordings were made from the closest distance possible, ca. 20–30 m, with a Sound Devices MixPre-3 or a Marantz PMD 661 MKII recorder and a Sennheiser ME 67/K6 (frequency response 40 Hz to 20 kHz) or Sennheiser MKH-70 directional microphone (frequency response 50 Hz to 20 kHz).

### Sound analysis for within-individual comparison

Overall, we carried out slightly enhanced versions of analyses previously used for the tambourine dove, presented in detail by^35^. All analyses were performed using Avisoft SASLab Pro software v. 5.3.00 (Avisoft Bioacoustics Germany^39^) and Raven Pro 1.6.x (Cornell Lab of Ornithology K. Lisa Yang Center for Conservation Bioacoustics^40^). The original recordings were taken with 48 kHz/24 bit sampling rate/quality, but because of SASLab Pro limitations, files in this study were downsized to 16-bit and were band-pass filtered from 150 to 800 Hz with a time-domain filter (FIR) to remove background noise.

First, we characterised whole songs using their duration (s), number of notes, and several frequency derivatives (Hz): peak frequency, lower quartile (25%), mean frequency (50%), upper quartile (75%), spectral centroid, minimum frequency, and maximum frequency (see Table S2 for details; Avisoft-SASLabPro manual^39^). Frequency measurements were taken with the One-dimensional transformation function/Amplitude spectrum (linear) feature of SASLab Pro and with a −18 dB threshold (resolution 0.046 Hz). Song durations and the number of notes were quantified based on selections performed in Raven Pro, which enabled a very restrictive visual inspection of each note. In the next step, we measured the characteristics of notes and pauses between notes within the song: peak frequencies of notes (later PF), note durations (later N), pauses between notes (later P), and pulse-to-pulse durations (later PTP; Fig. 1). The final characteristic was the time between the peak frequencies of adjacent notes. Here, these measurements are presented using a numbering convention with which, for example, PF_1_ and PF_2_ represent the peak frequency of the first and second notes, while PTP_1-2_ and PTP_2-3_ indicate the time between the maximum amplitudes of the first and second notes, and the second and third notes, respectively (see Fig. 1 for illustration). These measurements were derived from selections in Raven Pro, but with settings adjusted separately for frequency and time measurements, which gave the following resolutions: 23.4 Hz × 21.3 ms or 93.8 Hz × 5.33 ms, respectively. These ‘within-song’ measurements were used to determine if the within-song pattern of note separation is likely to be important for identity coding, as was found for tambourine doves^35^.

### Experimental protocol

#### Preparation of song stimuli

To prepare neighbour song stimuli, we recorded singing males 1–3 days before each experiment. Recordings were made from the closest distance possible, ca. 20–30 m, with a Sound Devices MixPre-3 or a Marantz PMD 661 MKII recorder and a Sennheiser ME 67/K6 or Sennheiser MKH-70 directional microphone. Recordings were made during birds’ daily active period, usually lasting until 4–5 h after sunrise (sunrise started 5:54–6:02). The exact locations of the singing males were determined with Garmin GPS 65 s receivers. Stranger stimuli were prepared with recordings from the local populations but collected at least 2 km from focal males. These recordings were collected opportunistically on a daily basis. Recordings were taken with 48 kHz/24 bit sampling rate/quality. The playbacks were digitally prepared to 82 ± 2 dB signal pressure level (at 1 m), which is the average natural amplitude of blue-headed wood-dove song (measured with a CHY 650 digital sound level meter; CHY Firemate Co., Ningbo, China). For both the treatment with neighbour and stranger stimuli, the highest quality song was randomly selected from the collection and duplicated 10 times over a period of 5 min (later PLAY) to prepare playback stimuli. All playback stimuli were created with Raven Pro 1.6.x and Avisoft SASLab Pro 5.2.

#### Experimental design and playback procedure

The playback experiment consisted of two trials, and each male was tested twice, once with the song of a neighbour, and once with the song of a stranger. The order of treatments for an individual was randomised. A neighbour was defined as an individual with a territory bordering that of the subject (not more than 250 m distant), whereas a stranger was defined as a random male from the local population whose territory was more than 2 km distant from the subject. Our observations suggest that a given male rarely sings from song posts separated by more than 250 m (usually less). Hence, a distance of > 2 km was a very conservative criterion for choosing strangers. In other words, the neighbours always stayed within range of their songs and had no other males between their territories. Strangers could not be heard from the focal male territory and there was a space between them and the focal male territory for at least few adjacent territories of other individuals. In case of trials with neighbour song, the one with better quality was chosen. In the case of stranger treatment, the song sample for playback was selected randomly from a list of males that included the geographical position of the recording (> 2 km distance), and recordings of the same individuals were never used more than once. Both treatments (neighbour and stranger stimuli) were performed on the same day between 05:50 and 11:30 (local time). The time between treatments ranged from 19 to 85 min (mean 29 ± 17.3), depending on how quickly the focal birds returned to their pre-stimulus behaviour (i.e., to singing from approximately the same song post and at the same rate as before the trial). In this way, we reduced the influence of potential natural interactions with real rivals between treatments and decreased the chance of testing different individuals (whose identity was checked based on their song pattern after the experiment). We used exclusively unique song recordings of different neighbours and strangers to avoid pseudoreplication^41^. We did not control for the presence of females or the stage of the breeding cycle, but all tested males were highly vocal. During each experiment, we simulated a situation in which a neighbour or stranger male appeared in the territory of the subject with a clear transgression of its boundaries. Before the experiment, one person placed a speaker (JBL Charge 4, Harman International Industries, Stamford, Connecticut, USA), connected to a Marantz PMD 661 MKII player, ca. 2 m above ground and approximately 50 m from the subject. In the second treatment, the loudspeaker was also placed ca. 50 m from the subject, but in a different location within the territory (but also from the side where the neighbour typically sang). Each trial lasted ≥ 11 min and consisted of three parts. In the first part of the trial, the male’s song was recorded for at least 1 min before the playback started. The main aim was to record at least 1 or 2 good-quality songs for later comparison with the response. During the second part of the trial (PLAY), the male’s behaviour was recorded for 5 min during the playback of 10 songs from the same male, either a neighbour or stranger, depending on the treatment. Song rate of playback reflected well the natural rate of singing.^37^ Then, in the POST playback phase of the experiments, the behaviour of males was recorded without any playback for the next 5 min. A similar approach has been used successfully with the same species, in playback experiments investigating recognition of conspecific and congeneric song^42^. Since we knew after the earlier study how the birds reacted to the song of stranger, and that they were completely ignoring the control songs of local tauraco species, we no longer did the control treatment.

During the experiments, we observed the subject's behaviour and recorded the songs he produced (Sound Devices MixPre3+ Sennheiser ME 67/K6 or Sennheiser MKH-70 directional microphones). A second observer was focused on the approaching response and was always placed to have a different viewpoint of the experimental scene. Both observers dictated their observations of bird behaviours, and tracks of recordings were later synchronised to obtain the timing of all activities. Synchronisation was done based on a high-frequency ‘ping’ sound generated at the beginning and end of the experiment (the ‘ping’ was far above the frequency of the focal species song and sounded natural, similar to small bird calls). For the vocal response, we characterised the number of songs produced by focal males during the PLAY and POST phases of the experiments. We also measured the latency of the approaching response to the speaker (s), the closest approach to the speaker (m), the amount of time spent within 25 m of the speaker, and the number of flights during the PLAY and POST phases.

### Statistical analyses

#### Individual differences in song

The main aim of the descriptive part of the study was to determine if a comparison of within-and between-individual song variation allows for individual recognition of blue-headed wood-doves, and which song characteristics are likely to serve in identity transmission. For this, we calculated Beecher's information statistic (*H*_*s*_) for entire song characteristics, first for song duration and frequency components separately and then for all the song characteristics together. Beecher's information statistic (*H*_*s*_) indicates the amount of information in a system that is available to convey individual identity. The *H*_*s*_ value is proportional to the number of individuals that can potentially be discriminated in a population^43^. All calculations were performed with the *calcHS* function of the R package 'IDmeasurer'^44^. We also calculated *H*_*s*_ for sequences of peak frequencies (PF) and pulse-to-pulse durations (PTP), to determine if the time–frequency pattern of initial notes is male-specific, as was found in the tambourine dove^35^. When the *H*_*s*_ was calculated for multiple variables, its value was presented for all variables at once, regardless of wheter they significantly contributed to distinguish individuals. Before we calculated *H*_*s*_ for multiply variables we also converted them into uncorrelated principal component with *calcPCA* function of the same package.^44^ Finally, for comparative purposes, we present the results of discriminant function analyses (DFAs) based on the same dataset to allow readers to associate *H*_*s*_ values with the efficiency of identity assignment for the study species. We conducted stepwise DFA implemented in IBM SPSS Statistics 28.0.1.0 using the measurements of whole song phrases and sequences of initial notes, which allowed for direct comparison with previous results on the song individuality of the tambourine dove^35^.

#### Playback experiment

Altogether, we measured seven variables that described the responses of males to playback. Because separate tests on the original variables would not be statistically independent or reveal the multivariate character of the response^45^, we combined all original variables into two orthogonal principal components (Table 1). For this, we used a principal component analysis procedure (PCA) with varimax rotation and Kaiser normalisation in IBM SPSS Statistics 28.0.1.0. We first ensured that the dataset was suited for such an analysis (Kaiser–Meyer–Olkin measure of sampling adequacy = 0.748, Bartlett test of sphericity = 89.04, *P* < 0.001). The first PC1 variable—Approaching—reflected the rapidity of the approach to the speaker during playback. Lower values of PC1 indicated that males performed many flights, quickly came close to the speaker, and stayed close for a longer time. The second component, PC2—After playback response—reflected the behaviour of the tested males after playback. Higher values of PC2 were associated with a higher number of flights and more songs during the POST phase of the experiments. The interpretation of PC1 is rather straightforward: lower values reflect a stronger response, as approaching a rival is a clear signal of increased aggression and readiness to fight. In the case of PC2, the component was linked with both song and flight activity after the end of the simulated intrusion. The number of flights during the POST period was never high (0–4) in comparison to the PLAY phase (0–13), while the ranges of song numbers produced during the PLAY and POST phases were the same (0–11). Therefore (together with what we observed in detail during the experiments), we would interpret higher values of PC2 as a faster return to the initial activity, i.e., returning to previous song post with a few flights to the previous song post and singing at a normal rate.

**Table 1:** Principal component loadings for blue-headed wood-dove responses to playback of neighbour and stranger songs.

Statistics and original response variables	PC1–Approaching	PC2–After playback response
Eigenvalue	3.12	1.36
% of variance	44.56	19.37
Cumulative %	44.56	63.93
Songs during playback	0.48	0.31
Songs after playback	0.39	**0.68**
Latency to the first flight (s)	**0.84**	**−**0.28
Closest distance to speaker (m)	**0.82**	**−**0.10
Time spent within 25 m to speaker (s)	**−0.75**	**−**0.23
Flights during playback	**−0.86**	0.11
Flights after playback	**−**0.21	**0.80**

Kaiser–Meier–Olkin = 0.748, Bartlett's test of sphericity χ^2^ = 89.037, *P* < 0.001. Values that makes a substantial contribution to the overall variance are in bold.

We tested for differences in the response to neighbour and stranger songs with generalised linear mixed-effect models (GLMM) using the 'lme4' package of R^46^. Our response variables were PC1 and PC2. The initial models included two main factors: (1) treatment (two levels: neighbour or stranger song), and (2) playback order (two levels: stranger first or stranger second). We included the first-order interaction terms and used male identity as a random effect. In subsequent models, we tested if the frequency of the song of the focal male, as well as that of the song used for playback, affected response strength. We recalculated the mixed models described above with additional independent continuous variables describing focal male frequency, playback frequency, and relative frequency (i.e., difference between focal male and playback frequency).

We checked the models' assumptions using the 'DHARMa' package of R^47^ and found that they were not over-dispersed (all *P* ≥ 0.728); visual inspection of Q-Q plots confirmed the normality of residuals (Kolmogorov–Smirnov tests *P* ≥ 0.198). We present only the best-fitted models, based on the lowest values of AIC. All *P* values reported are two-tailed.

### Ethical note

To our knowledge, the individuals tested in the experiment reflected the population in a representative way with no potential biases resulting from social background, self-selection, habituation, or other factors as indicated in the STRANGE framework^48^. This study was designed and performed in accordance with the ARRIVE guidelines^49^. Our experimental procedure adhered to the ASAB/ABS Guidelines for the care and use of animals (The Ethics Committee (ASAB) and the Animal Care Committee (ABS), 2019) and was approved by all responsible local bodies listed below as well as by the Polish Laboratory Animal Science Association (certificate no. 1952/2015 to TSO, conforming to Directive 2010/63/EU). Our experimental procedures were approved in Ghana by the Forestry Commission (Wildlife Division), permit no. WD/A.185/Vol.13/80.

## Results

### Within- and between-individual song variation

**Figure 2:**
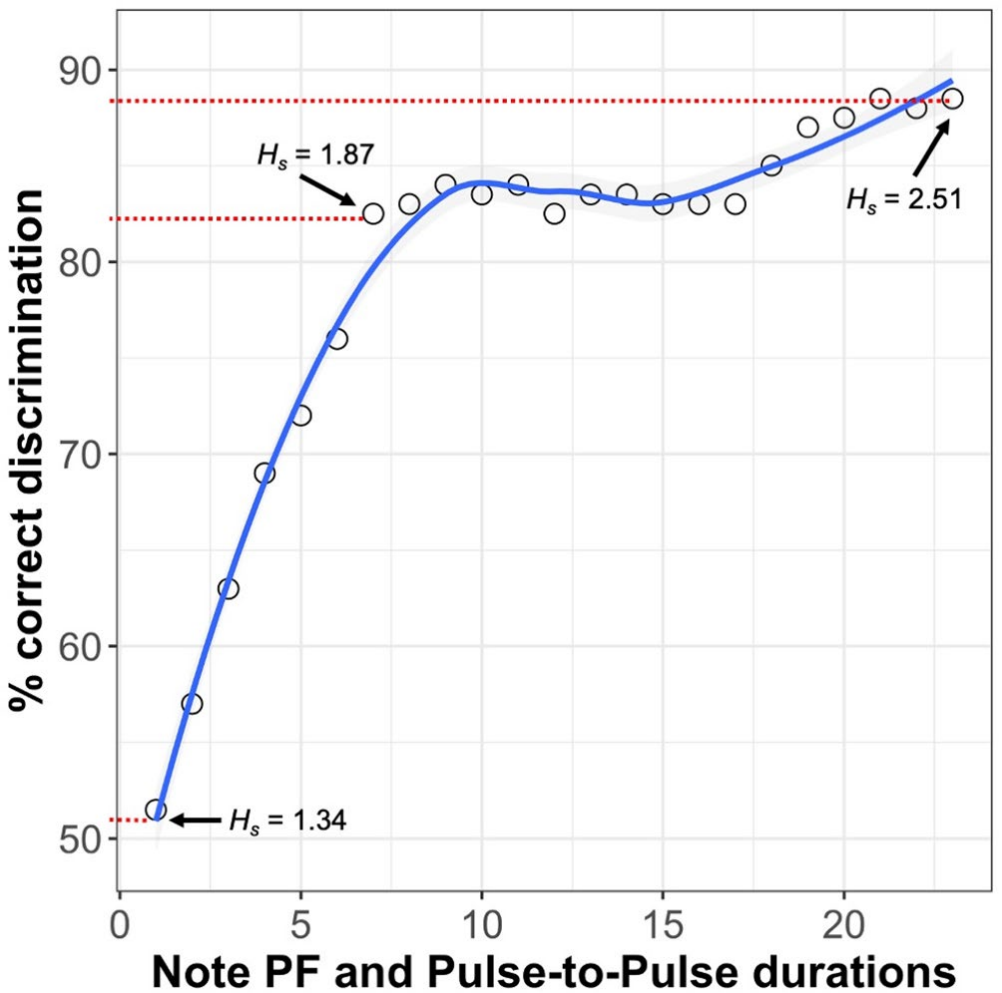
Percentage of correct classification of the blue-headed wood-dove song based on discriminant function analysis (DFA) with note peak frequency (PF) and pulse-to-pulse duration (PTP). Numbers on X-axis indicate sequences analysed with DFAs: 1—PF_1_ + PTP_1–2_, 2—PF_1_ + PF_2_ + PTP_1–2_ + PTP_2–3_ and so on. *H*_*s*_*—*Beecher's statistics for the three chosen sequences of PF + PTP. The red dotted lines indicate the data points chosen to showcase the values.

Detailed data of bioacoustics measurements of songs used to estimate identity coding potential are presented in Table S1. Some, but not all, of our measurements of song characteristics—as analysed using ‘IDmeasurer’ and Beecher’s statistic *H*_*s*_—revealed a potential for identity coding in the study species. The single whole song variables did not seem to convey a large amount of information regarding individual identity (*H*_*s*_ values between 0 and 1.27; Table 2). Likewise, time-related variables (either together or separately) and bandwidth appeared to be completely irrelevant for individual recognition (Table 2). On the other hand, when we calculated *H*_*s*_ for all frequency characteristics together or for all frequency and time characteristics, there was clearly a large potential for identity coding (*H*_*s*_ = 2.48). Even higher values of Beecher's statistic were obtained when we analysed values of peak frequencies and pulse-to-pulse duration (see Fig. 2), up to *H*_*s*_ = 2.51 for the longest possible sequence.

**Table 2:** Comparison of *H*_*S*_ (Beecher’s statistics) calculated for single, grouped (time, frequency) or all (time and frequency) song parameters.

Song characteristics	*H*_*S*_ for single variables	*H*_*S*_ for pooled time or frequency parameters	*H*_*S*_ for all time and frequency parameters
Time related parameters			
Duration	0	0.79	
Number of notes	0.5		
Frequency parameters			
Peak frequency	1.16	2.02	2.48
Lower quartile	1.06		
Mean frequency	1.07		
Upper quartile	1.07		
Spectral centroid	1.0		
Minimum frequency	1.27		
Maximum frequency	0.76		
Bandwidth	0		

*H*_*S*_ indicate the amount of information in a system, the higher value the larger potential for identity coding.

Next, we applied the methodology from a previous analysis conducted for the tambourine dove^35^ to classify songs to different individuals using stepwise DFAs. Based on the most important measurements of whole songs (song duration; number of notes; minimum, maximum, and mean peak frequency), DFA correctly classified individuals in 88.5% of cases in a leave-one-out classification. When we replicated DFAs based on note and pause sequences (PF + PTP)^35^, we found that songs were correctly classified 51.5 to 82.5% of cases (sequences contained 1 to 12 notes, which reflected the longest sequence for the tambourine dove measured in^35^. This value increased to 88.5% for analyses of PF_1_ to PF_23_ and PTP_1-2_ to PTP_22-23_ (Fig. 2). Figure 2 presents values of *H*_*s*_ for a few sequences of PF + PTP measurements, which enables direct comparison between *H*_*s*_ values and the efficiency of discriminant analysis. Both sets of results indicate that differences in the distribution of notes in time create the potential for identity coding.

### Response to neighbour and stranger songs

**Figure 3:**
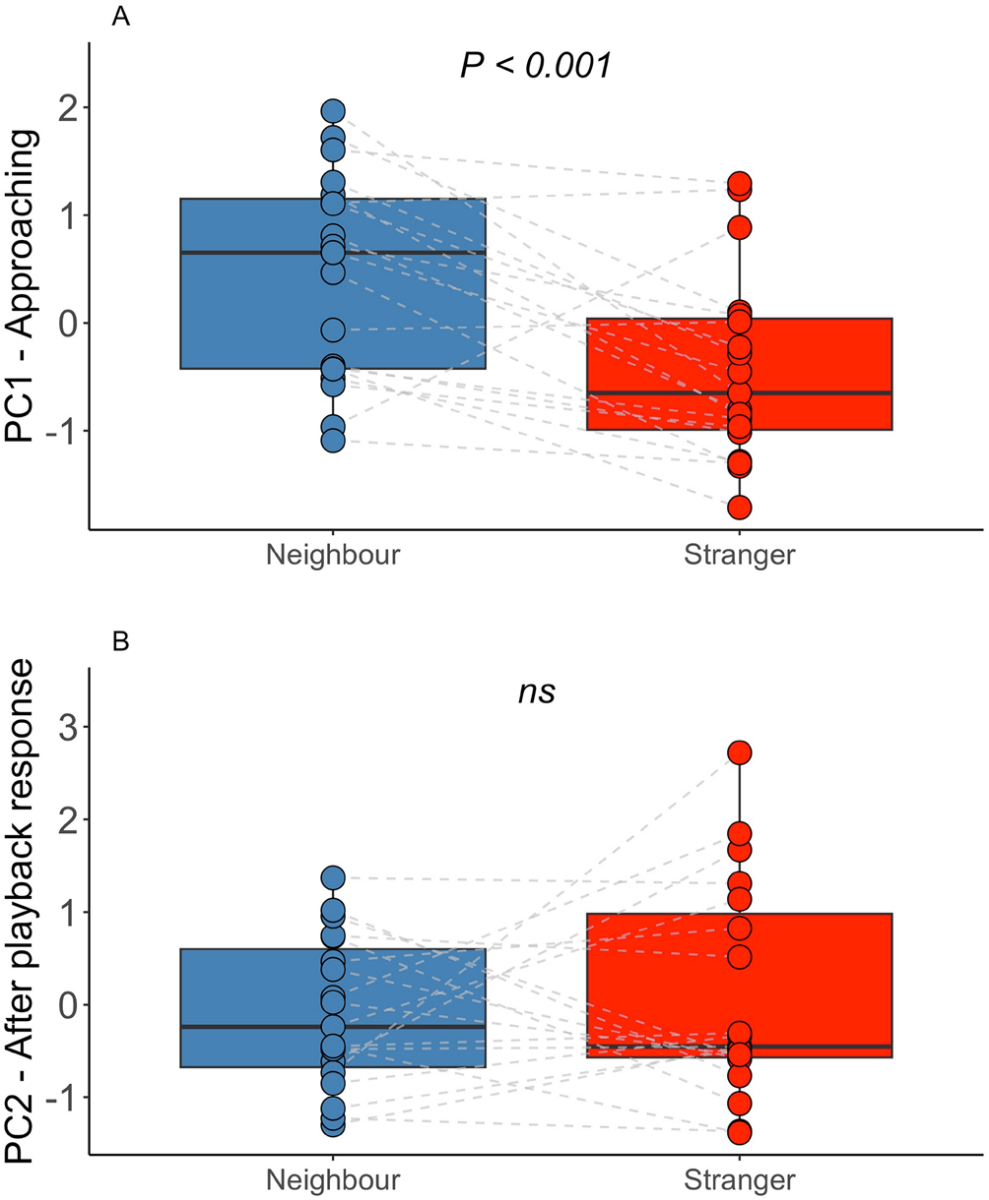
The blue-headed wood-dove male responses to playback of neighbour and stranger songs measured with PC1-approaching and PC2-after playback response compound response measurements (*N* = 19). The dotted lines represent the same tested male responding to neighbour and stranger songs.

We used playback experiments to determine whether blue-headed wood-dove males (*N* = 19) are able to distinguish between the songs of a familiar neighbour and an unfamiliar stranger. When birds were presented with the two types of playback, we observed significant differences in PC1, the approaching response (Table 3, Fig. 3A), but not in PC2, the after-playback response (Table 4, Fig. 3B). Males responding to stranger playback approached the speaker more quickly, came nearer to it, and stayed close to it for a longer time. The significant Treatment × Order interaction indicated that males responding for the first time to playback of the stranger approached the speaker faster and closer (Table 3). The difference noted in the approaching response also reflected the fact that birds performed more flights in response to strangers’ songs during the playback phase of the experiments (Fig. 3A).

**Table 3:** The best (lowest AICc) model explaining variation in PC1-approaching response compound measure of response to playback.

	Estimate	SE	z value	Pr( >|z|)
(Intercept)	1.48	0.37	4.02	0.0004
Treatment	−0.84	0.21	−4.01	**0.000897**
Order	−0.45	0.21	−2.17	**0.044**

The fixed effects in our models were treatment (neighbour or stranger), order of playback and interaction between treatment and order. We included male ID as a random effect. Significant effects are in bold.

**Table 4:** The best (lowest AICc) model explaining variation in PC2-after playback response compound measure of response to playback.

	Estimate	SE	z value	Pr( >|z|)
(Intercept)	−0.27	0.50	−0.54	0.597
Treatment	0.18	0.31	0.57	0.576

The fixed effects in our models were treatment (neighbour or stranger), order of playback and interaction between treatment and order. We included male ID as a random effect.

As mentioned above, we did not control for the frequency of the playback song, which varied among the individuals from whom the songs had been recorded. To investigate if this factor may have had an effect, we built mixed models in which we included the song frequency of focal males, the frequency of the playback, and the relative difference between the two (playback minus focal song pitch). We used mean frequency instead of peak frequency because the mean was less susceptible to deviations due to recording quality, and for the focal males in the experiments, we did not always have many songs to measure (if males did not vocalise very much). However, the correlation between the mean and peak frequency of a given good-quality song was very high (correlation *r* = 0.97, *N* = 200, *P* < 0.001).

**Figure 4:**
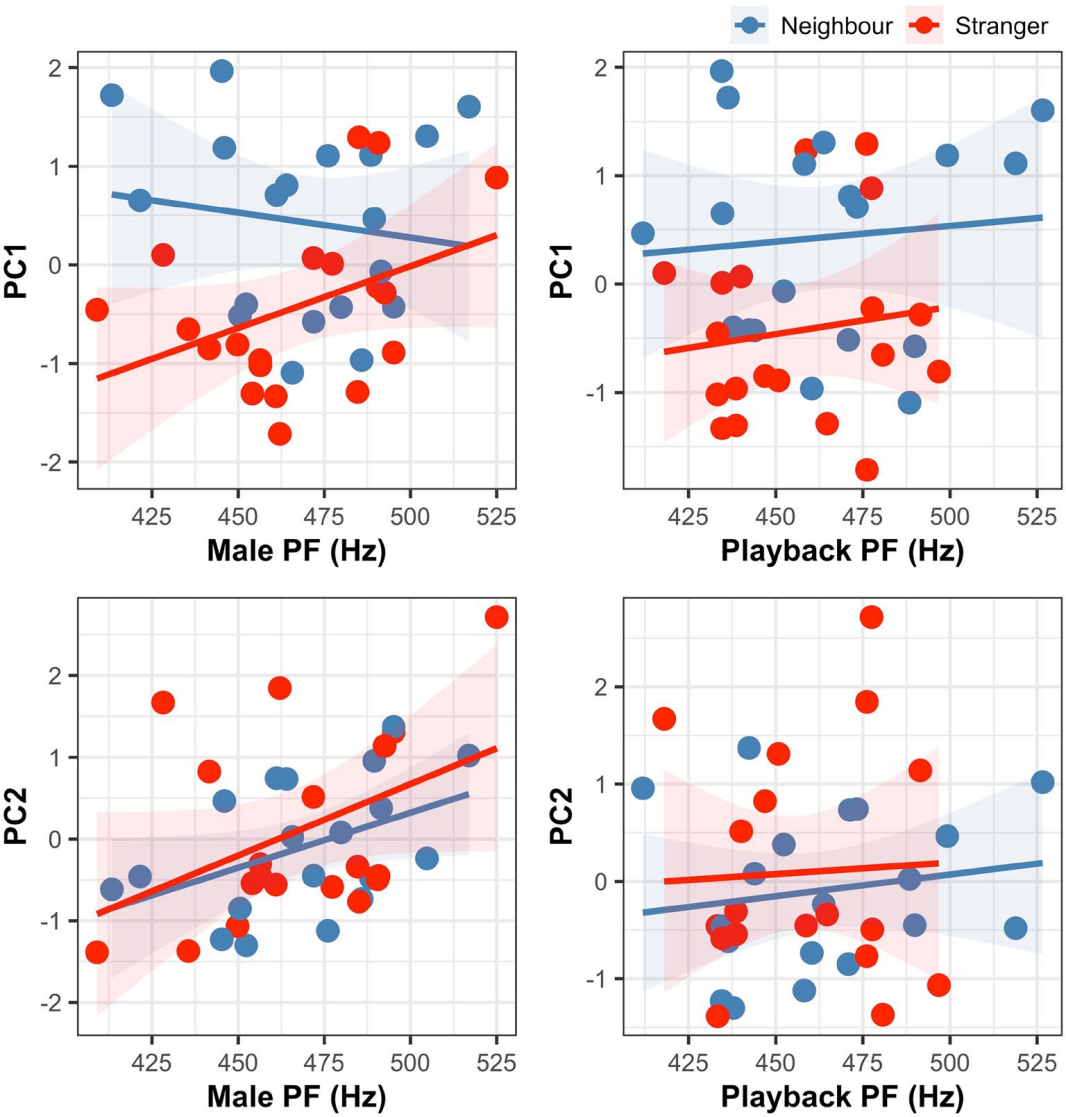
Relationships (linear regression models) between response to playbacks (measured with PC1-approaching and PC2-after playback response) and peak frequencies (PF) of focal male and playback songs. Different colours indicate neighbour and stranger treatments (*N* = 19 males tested twice).

Neither the song frequency of the focal male nor that of the playback had a significant effect on the approaching response (PC1), and none of the mixed models that incorporated song frequency were any better (i.e., lower AIC) than the model presented in Table 3. In the case of the PC2 response, we detected a statistically significant effect of the focal male frequency, which greatly improved the initial model (Table 5). Specifically, males singing with a higher frequency had higher values of PC2 responses, meaning that after the playback, they returned more quickly to their previous singing activity and left the vicinity of the speaker. Males who sang lower-frequency songs stayed closer to the speaker for a longer time in the POST phase without singing (Fig. 4). There was a significant Treatment × Order interaction in the final model (Table 5) that indicated that males responding for the first time to the playback returned more quickly to their previous singing activity (higher PC2) than in the second trial with the same individual.

**Table 5:** The best (lowest AICc) model explaining variation in PC2-after playback response compound measure of response to playback including song frequency of the focal males.

	Estimate	SE	z value	Pr( >|z|)
(Intercept)	−8.69	2.78	−3.12	0.00516
Treatment	−0.61	0.44	−1.37	0.179
Order	−2.15	1.09	−1.98	0.064
Focal male frequency	0.020	0.006	3.28	**0.00372**
Treatment × order	1.50	0.70	2.15	**0.04894**

The fixed effects in our models were treatment (neighbour or stranger), order of playback and interaction between treatment and order. The focal male frequency was included in the model as a continuous explanatory variable and male ID as a random effect. Significant effects are in bold.

## Discussion

By analysing the songs of numerous male blue-headed wood-doves, we discovered that there is a large potential for identity coding in this species. We were then able to demonstrate experimentally that these birds are able to discriminate between the songs of neighbours and strangers. To the best of our knowledge, this study is the first to report NSD in the order Columbiformes and is one of only a few studies showing that NSD occurs in a non-learning bird species.

### Individuality of song

We observed that the song of the blue-headed wood-dove is individually specific and that the highest potential for identity coding is found in the unique patterns of note sequences within songs rather than single song characteristics such as frequency or duration. This individual specificity is visually evident from the spectrograms of different individuals (Fig. S1), but more importantly, it was statistically supported by different types of analyses. This result was unsurprising as a similar pattern of individual variation was reported in the closely related tambourine dove^35^. Both species inhabit acoustically similar habitats; hence, similar rules for coding and maximising song transmission should be expected. Despite the similarities between the two species, though, they are easily distinguishable based on different patterns of note timing within the song^37^. In the blue-headed wood-dove, pauses between notes are initially longer and then consistently shortened as the song phrase progresses. In the tambourine dove, pauses in the initial section follow an individually specific pattern and result in the grouping of two or three notes in a distinct series (details in^35^). The two species also differ in size and weight, with the blue-headed wood-dove being much larger (body mass ~ 64% larger) than its congener^50^. It is interesting, however, that the frequencies of their songs are very similar (min–max: 351–562 Hz for blue-headed and 246–551 Hz for tambourine dove; own. unpubl. data), with the smaller species having a slightly lower frequency. Similar interspecific differences were observed for the pink pigeon *Nesoenas mayeri* and the Madagascan turtle dove *Neosoenas picturata*^51^, and overall this suggests that, at least in this respect, their songs may be optimised for habitat propagation^52^. What is most important in the context of this study is that there is undoubtedly a strong acoustic basis for individual recognition in the study species: within-individual song variation is minimal compared to differences between individuals. The overall acoustic simplicity of its song makes the blue-headed wood-dove an ideal model for experimental testing on identity coding using song manipulation and artificially synthesised songs.

### Neighbour-stranger discrimination

In blue-headed wood-dove males, the main response axis that differentiated between the response to neighbour and stranger playback was the approach to the speaker. This is quite typical for NSD experiments (e.g.,^53^, including those focused on non-passerines^19,26^. When birds approach faster and closer to playback from a potential stranger than from a known neighbour, it is viewed as support for the "dear enemy" hypothesis. This pattern of approach is a good proxy of increased aggression and predicts an escalation to an attack^54^.

In the case of the vocal response to the playback of neighbour and stranger songs, it is difficult to detect clear differences, at least at first glance (Table 4, Fig. 3B). Observations of singing males of this species indicate that they adopt a special posture and focus solely on this activity (own unpublished observations). Because song phrases are long, the performance of the song takes time (Table S1). Males rarely produce incomplete songs consisting of only a few (1–4) initial notes, which suggests that once they commit to song production, it is hard for them to stop. Therefore, when a male detects the intrusion of a potential rival from a distance, he most likely has to choose between remaining still and singing from the same position or moving closer to the rival without singing. In this scenario, the number of songs produced during the PLAY phase would be inversely related to the promptness of the approaching response: the faster a male decides to approach, and to try to locate a rival by flying, the less time remains for singing. The males with the strongest response performed their first flight after the first song of the playback and made flights after every, or almost every, song played by the speaker. Hence, approaching during playback is unambiguously the stronger response.

Interpretation of the vocal response is challenging but possible if we keep in mind that it is related to the above-mentioned "mechanics" of responding—namely, it is not possible to sing and approach simultaneously. Analyses that included both approaching and vocal responses revealed more individual variability in the response. Again, such a response pattern is not unusual and has been found in other birds such as the blue grouse (*Dendragapus obscurus*)^19^. Moreover, in bird species that interact with rivals using both songs and calls, the stronger response is often connected with a switch from singing (longer vocalisation) to calling (shorter vocalisation)^20,53^.

If focal males maintain the same level of singing during playback as before, it essentially guarantees that they are not moving. In this case, they are still signalling territory ownership but not escalating the conflict. It is less straightforward to decode the meaning of observations in which a male ceased singing but did not approach. Such behaviour could be interpreted as a more cautious response, for example, toward a rival perceived as too strong to respond by approaching^55^. However, it could also be interpreted as a response to a rival viewed as threatening enough to merit attention but not so much that he must be chased away. In practice, when experimenting in tropical forests, we must also always consider the possibility that the focal male did approach the speaker, but we overlooked the flight. We hope that we had no such cases here as the experiments were conducted by two experienced observers who have worked previously with this study species. Regardless, it is also extremely important to link the vocal response during playback with the location of singing after playback. Here, our principal component analysis enabled us to extract variables related to approaching (PC1) and to the behaviour (both vocal and movement) after playback (PC2). Although we did not find a significant effect of the treatment or the order of playbacks on PC2 (Table 4), the addition to the model of the song frequency of the focal male revealed an interesting pattern that sheds light on the factors that shape the response to rivals in this species (Table 5). We found that males who sang lower-frequency songs spent more time during the POST phase singing from a stationary location. In most cases, they were sitting in the last place in which they were found during playback, and it appeared as if the males were waiting to see if the intruder would sing again. Instead, the focal males who sang with a higher frequency came back to their starting positions more quickly and resumed singing from their pre-playback song posts.

To interpret these results, we need to know what factors affect song frequency and what kind of information it may carry. Between-species comparisons usually generate negative correlations between body size and the frequency^56^. This appears to be a general rule for various animal taxa, despite subtle differences related to the evolutionary history of taxa or how measurements of both, body size and song frequency were taken.^57,58^ This is explained by the theoretical prediction that larger-sized structures for vibrating and resonating are more effectively producing and coupling lower frequencies to the medium^2^. Theoretically, this pattern of body size–signal pitch allometry should be apparent also within a species. However, it is much less supported by research; some studies found the expected negative correlation, while other did not or even identified a reversed relationship (reviewed in^36,59^). Likely, small songbirds, with their pronounced vocal abilities and the relatively small variability in adult body size, may invalidate this size-pitch relationship . Nevertheless, even for songbirds, when body size parameters correlate negatively with song pitch, the frequency of playback stimuli affects birds’ responses. In an experiment on willow warblers (*Phylloscopus trochilus*), males responding to relatively low-pitched songs (compared to those of the subject) stayed farther away from the speaker but responded more actively^59^. This pattern was explained as reflecting the conflicting motivation of the tested males, who were threatened by the low-pitch songs of larger intruders but still wanted to chase off an intimidating rival. In the blue-headed wood-dove, songs are usually produced with a fixed frequency within individuals, and larger males produce lower-frequency songs (own unpublished data). Here, the song frequency used in playback had no effect on either the PC1 or PC2 responses, which indicates that the primary factor shaping the response to a perceived intrusion is the identity of its source (neighbour vs stranger). However, the behaviour of males after playback was significantly affected by their own song frequency (Table 5, Fig. 4). The pattern we found—males with lower-pitch song stayed closer to the speaker for longer without singing—suggests that larger males may have been more motivated to locate a rival and initiate a physical contest. Instead, males with higher-pitch songs quickly returned to their initial song posts after the playback stopped, and started singing again. It was interesting that we did not detect any effect of the playback song pitch per se or the relative difference in pitch (playback minus focal song pitch). This suggests that, when it comes to making decisions about the intensity of defence, the self-assessment of the territory holder is very important. However further experiments with manipulation of rivals’ song pitch are necessary for making general conclusions.

Because our experiment was designed primarily to test NSD, not the effect of song frequency, we did not choose songs for playback based on their frequency. For the neighbour treatment, such a choice would be inherently limited by the number of neighbours (and their song frequencies), while for the stranger treatment, a wider range of song frequency variation would be possible (we had a surplus of strangers recorded). In the future, it would be interesting to conduct further experiments that manipulate song frequency so that experimental males are presented with intruders singing with the minimal or maximal frequency found in this species. Such an approach should enable the direct assessment of the function of song frequency in an agonistic context.

## Conclusions

Our results are consistent with Fisher's "dear enemy phenomenon"^8^ and suggest that blue-headed wood-doves use NSD to keep the cost of territorial defence at a reasonable level. Everything that is currently known about territoriality in this species, including our own observations and recordings in the same locations (between Jan 2019 and Dec 2022), supports a strong attachment to a site, likely life-long (own unpublished data). Such long-term territory tenure in birds is often based on well-established relations with neighbours, for which individual recognition is fundamental^60^. In this context, a strong approaching response to a stranger is not surprising, as this kind of intruder would constitute a stronger threat to the territory holder. Our findings suggest that, as in many songbirds^38^, blue-headed wood-doves base their initial decisions about territory defence on song, and tune their response using additional information about rival identity from acoustic stimuli. Extraction of information on body size, based on the comparison of the own and rival song pitch, seems to be possible but must be proven in a separate experiment.

### Supplementary Information

Supplementary Figure S1.

Supplementary Table S1.

Supplementary Table S2.

## Data Availability

The datasets analysed during the current study are available from the corresponding author on reasonable request (Tomasz S. Osiejuk, email: osiejuk@amu.edu.pl).
