# Supplementary material for: Neighbour–stranger discrimination in an African wood dove inhabiting equatorial rainforest
Source: Sci Rep. 2024 Feb 21;14:4252. doi: 10.1038/s41598-024-53867-7 (PMC10879109; doi:10.1038/s41598-024-53867-7)

**Figure S1.** Spectrograms illustrating songs of three different blue-headed wood-dove males (colours indicate different individuals for easier comparison of individual differences).


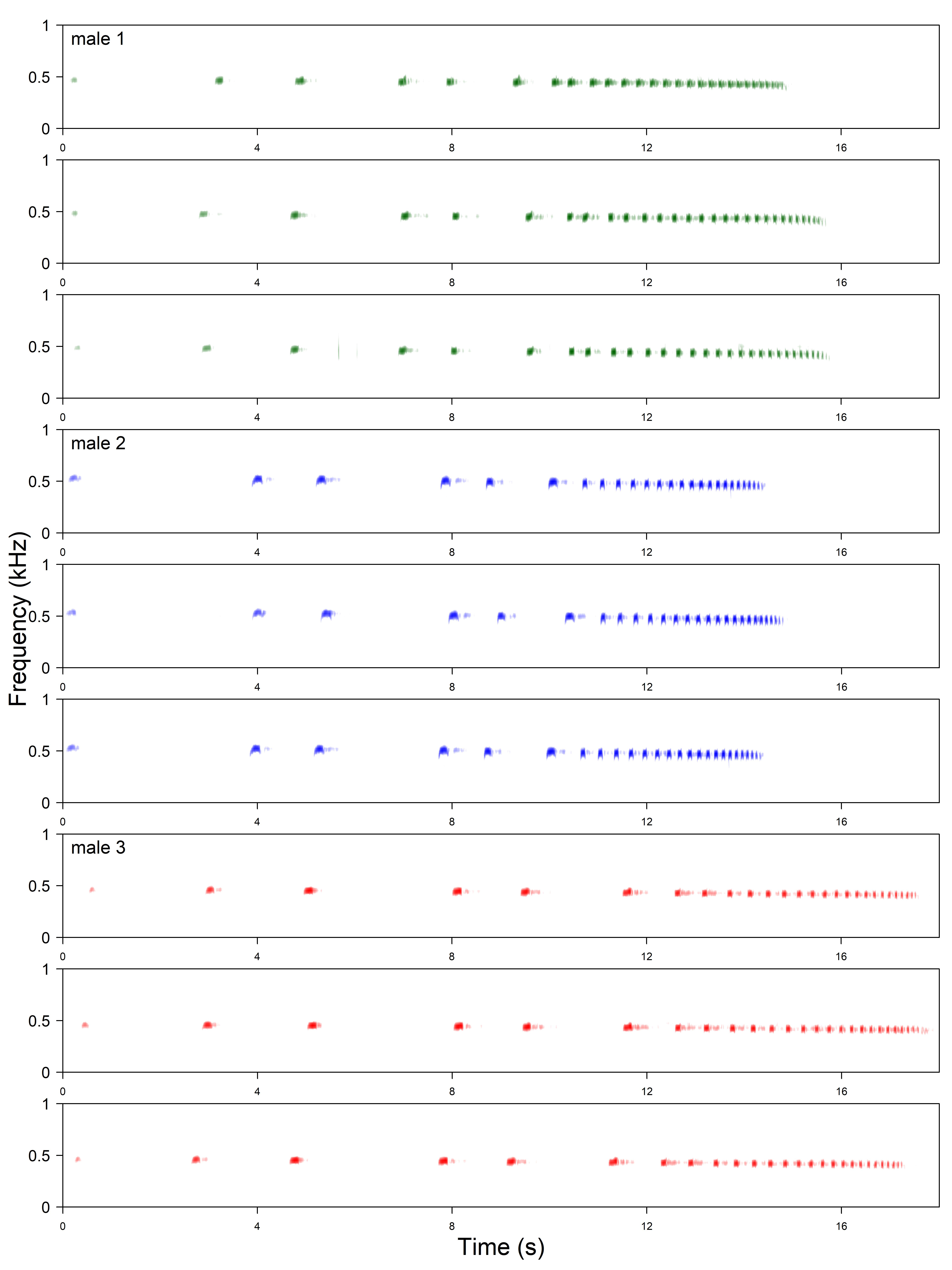

Supplement: Supplementary file 1 — Supplementary Figure S1. [file 41598_2024_53867_MOESM1_ESM.docx]
