# Supplementary material for: Neighbour–stranger discrimination in an African wood dove inhabiting equatorial rainforest
Source: Sci Rep. 2024 Feb 21;14:4252. doi: 10.1038/s41598-024-53867-7 (PMC10879109; doi:10.1038/s41598-024-53867-7)
Supplement: Supplementary file 2 — Supplementary Table S1. [file 41598_2024_53867_MOESM2_ESM.docx]

**Table S1. Summary of the results of bioacoustics measurements (mean±sd) for 10 individuals used for the evaluation of the potential of identity coding.**

| Male (N song sample) | Song duration (s) | Number of notes | Peak frequency (Hz) | Lower frequency quartile (Hz) | Mean frequency (Hz) | Upper frequency quartile (Hz) | Spectral centroid (Hz) | Minimum frequency (Hz) | Maximum frequency (Hz) | Bandwidth (Hz) |
| --- | --- | --- | --- | --- | --- | --- | --- | --- | --- | --- |
| 1 (17) | 15.6±1.16 | 30±0.8 | 440±4.8 | 432±1.7 | 443±3.3 | 472±47.6 | 453±28.8 | 417±2.6 | 469±5.6 | 52±6.8 |
| 2 (13) | 12.9±1.46 | 27±1.8 | 489±12.8 | 467±4.5 | 487±4.2 | 505±4.2 | 476±4.1 | 440±6.6 | 525±4.7 | 85±7.5 |
| 3 (33) | 18.0±1.04 | 32±0.7 | 482±5.0 | 465±5.1 | 482±2.8 | 495±5.5 | 471±4.8 | 450±4.7 | 518±7.3 | 68±8.3 |
| 4 (15) | 18.4±1.03 | 27±1.2 | 498±13.1 | 481±5.7 | 495±6.7 | 507±7.1 | 491±5.5 | 458±4.1 | 523±6.4 | 66±6.4 |
| 5 (21) | 14.6±1.62 | 25±0.9 | 459±6.4 | 443±6.4 | 442±2.6 | 458±2.5 | 455±2.0 | 424±3.1 | 488±5.1 | 65±5.7 |
| 6 (14) | 16.4±1.47 | 25±0.8 | 463±6.5 | 445±1.8 | 461±1.7 | 461±1.7 | 457±2.0 | 422±1.3 | 486±2.6 | 64±3.1 |
| 7 (30) | 13.8±2.41 | 25±0.9 | 443±6.4 | 431±1.7 | 444±3.0 | 454±4.9 | 444±2.8 | 412±3.4 | 471±6.8 | 59±8.3 |
| 8 (10) | 18.2±1.68 | 29±1.5 | 429±8.2 | 415±1.7 | 429±1.9 | 440±1.6 | 425±3.7 | 398±2.0 | 456±3.3 | 57±3.8 |
| 9 (17) | 18.4±2.03 | 31±1.1 | 477±6.9 | 471±3.7 | 485±5.8 | 502±8.4 | 483±6.3 | 483±6.3 | 531±22.8 | 85±24.1 |
| 10 (30) | 15.9±1.1 | 27±0.7 | 423±6.0 | 414±3.1 | 426±3.3 | 441±3.7 | 429±3.1 | 392±3.2 | 459±6.9 | 67±6.9 |
| Summary of the entire dataset (10 males, 200 songs) | | | | | | | | | | |
| Grand mean±sd | 16.2±2.02 | 28±2.4 | 460±25.8 | 447±23.8 | 461±25.3 | 476±25.5 | 458±21.9 | 426±22.1 | 493±29.3 | 67±10.1 |
| Min–Max | 8.5–23.6 | 23–33 | 413–520 | 410–491 | 421–509 | 434–625 | 419–525 | 388–463 | 452–597 | 43–154 |
