# Supplementary material for: Neighbour–stranger discrimination in an African wood dove inhabiting equatorial rainforest
Source: Sci Rep. 2024 Feb 21;14:4252. doi: 10.1038/s41598-024-53867-7 (PMC10879109; doi:10.1038/s41598-024-53867-7)
Supplement: Supplementary file 3 — Supplementary Table S2. [file 41598_2024_53867_MOESM3_ESM.docx]

Table. S2

| **Characteristics of song** | **Abbreviation  (if used)** | **Definition** |
| --- | --- | --- |
| Note | N or N_x_ where subscript reflects number of note within song phrase | a continuous line on a spectrogram, which is also referred to as an element; called element; in the case of the studied species, notes are heard as separate sounds, hence, are also equivalent to syllables in other species |
| Pause | P or P_x_ (as above) | duration of time between neighbouring notes; |
| Song phrase |  | the entire sequence of notes building the song, usually repeated with very stereotyped manner and with gaps between phrases much longer than between notes within phrases |
| Duration (s) |  | total time of the song phrase, i.e. from the beginning of the first note to the end of the last note |
| Number of notes |  | total number of notes within song phrase |
| Peak frequency of notes (Hz) | PF or PF_x_ (as above) | the frequency of the maximum amplitude of a single note spectrum |
| Pulse-to-pulse duration (s) | PTP or PTP_x-y_ (as above) | time between peak frequencies of adjacent notes |
| **Frequency spectrum characteristics of the entire song phrases^1^** | | |
| Peak frequency (Hz) | the frequency of the maximum amplitude of the spectrum (where spectrum is measured for the whole song phrase) | |
| Lower quartile (Hz) | the frequency below which 25% of the total energy of the spectrum is located | |
| Mean frequency (Hz) | the frequency below which 50% of the total energy of the spectrum is located | |
| Upper quartile (Hz) | the frequency below which 75% of the total energy of the spectrum is located | |
| Spectral centroid (Hz) | the weighted mean of the spectrum with the magnitudes as the weights | |
| Minimum frequency, Maximum frequency (Hz) | frequencies at which the amplitude falls short of the maximum of the spectrum minus the threshold; the threshold used in our measurements was set to -18 dB (see figure below) | |
| Bandwidth (Hz) | the difference between the maximum and minimum frequency | |

^1^More details presented here: <http://www.avisoft.com/Help/SASLab/menu_curve_display_spectral_characteristics.htm>


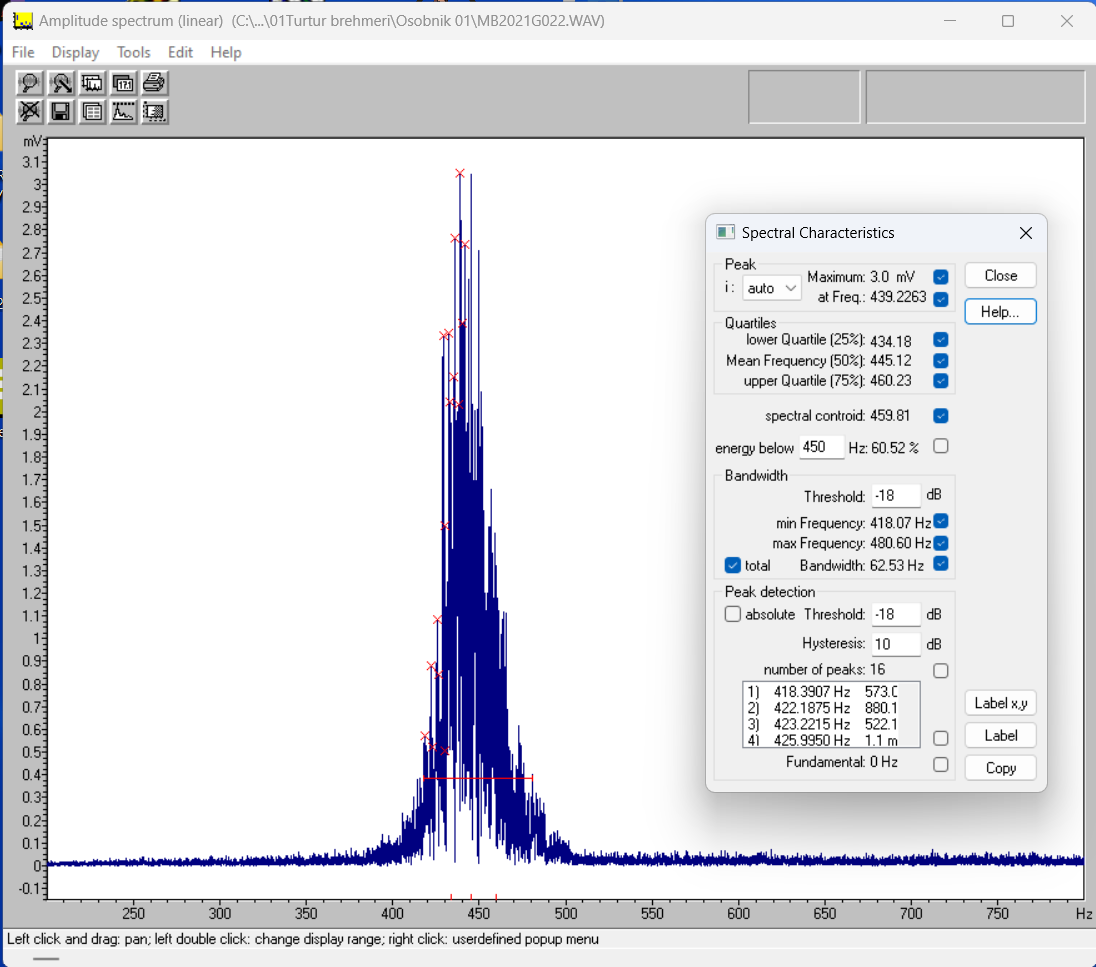


The amplitude spectrum window illustrates frequency spectrum measurements taken in Avisoft SAS Lab Pro.
